# Supplementary material for: A Convenient Approach towards the Synthesis of ADMDP Type Iminosugars and Nojirimycin Derivatives from Sugar-Derived Lactams
Source: Molecules. 2021 Sep 8;26(18):5459. doi: 10.3390/molecules26185459 (PMC8464940; doi:10.3390/molecules26185459)
Supplement: Supplementary file 1 [file molecules-26-05459-s001.zip › molecules-1338349-supplementary.pdf]

A convenient approach towards the synthesis of ADMDP type iminosugars and nojirimycin derivatives from sugar-derived lactams.

Piotr Szcześniak, Barbara Grzeszczyk and Bartłomiej Furman*

Institute of Organic Chemistry, Polish Academy of Sciences

Kasprzaka 44/52, 01-224 Warsaw, Poland

Corresponding authors: bartlomiej.furman@icho.edu.pl

Table of Context:

Copies of ^{1}H-, ^{13}C-NMR, spectra for new compounds: **3a, 2-*epi*-3a, 3b, 6a, 6b, 6c**. *S1-S12*

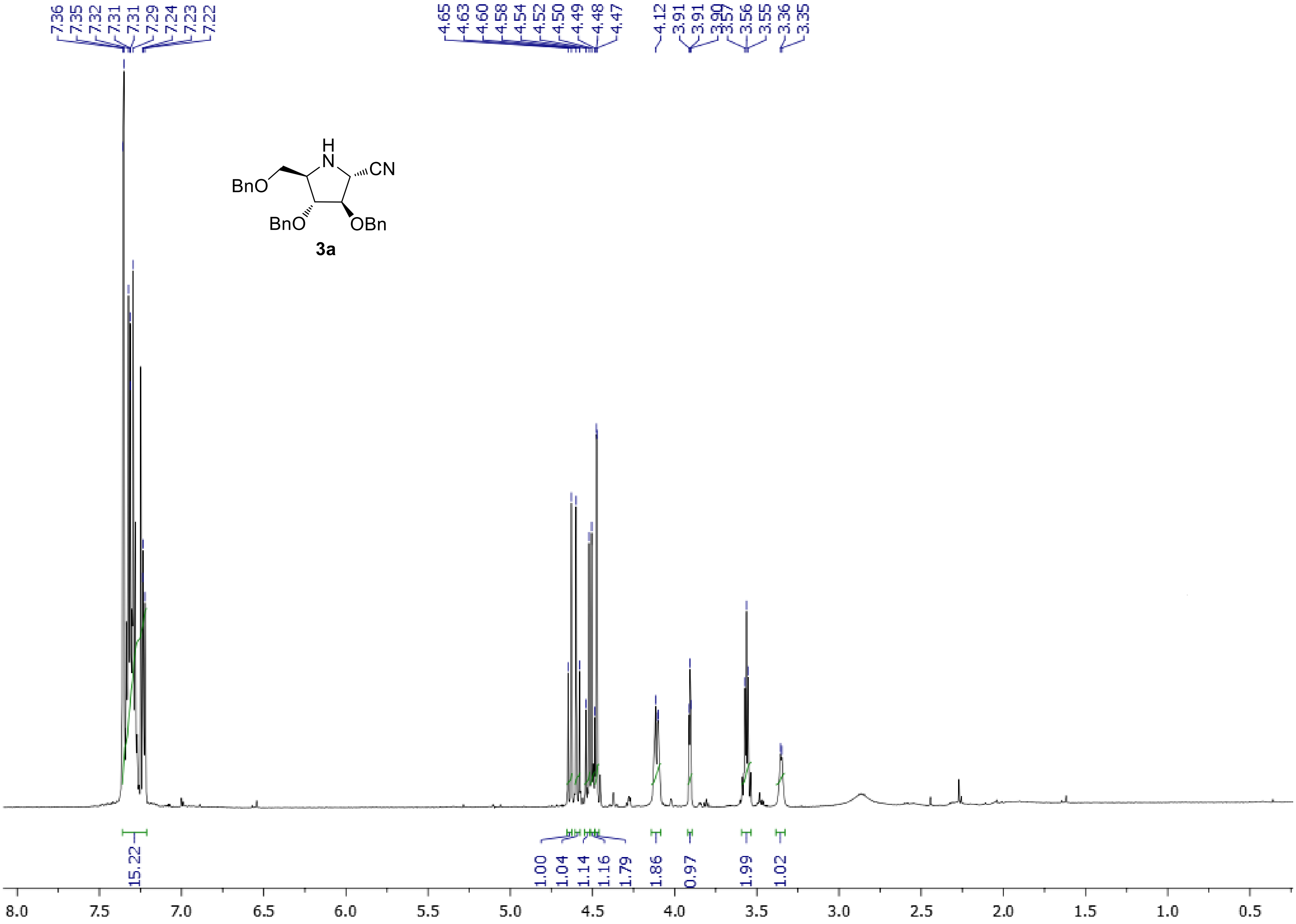
7.36
7.35
7.32
7.31
7.31
7.29
7.24
7.23
7.22
4.65
4.63
4.60
4.58
4.54
4.52
4.50
4.49
4.48
4.47
4.12
3.91
3.91
3.90
3.57
3.56
3.55
3.36
3.35
H
N
CN
BnO
BnO
OBn
3a
15.22
1.00
1.04
1.14
1.16
1.79
1.86
0.97
1.99
1.02
8.0
7.5
7.0
6.5
6.0
5.5
5.0
4.5
4.0
3.5
3.0
2.5
2.0
1.5
1.0
0.5

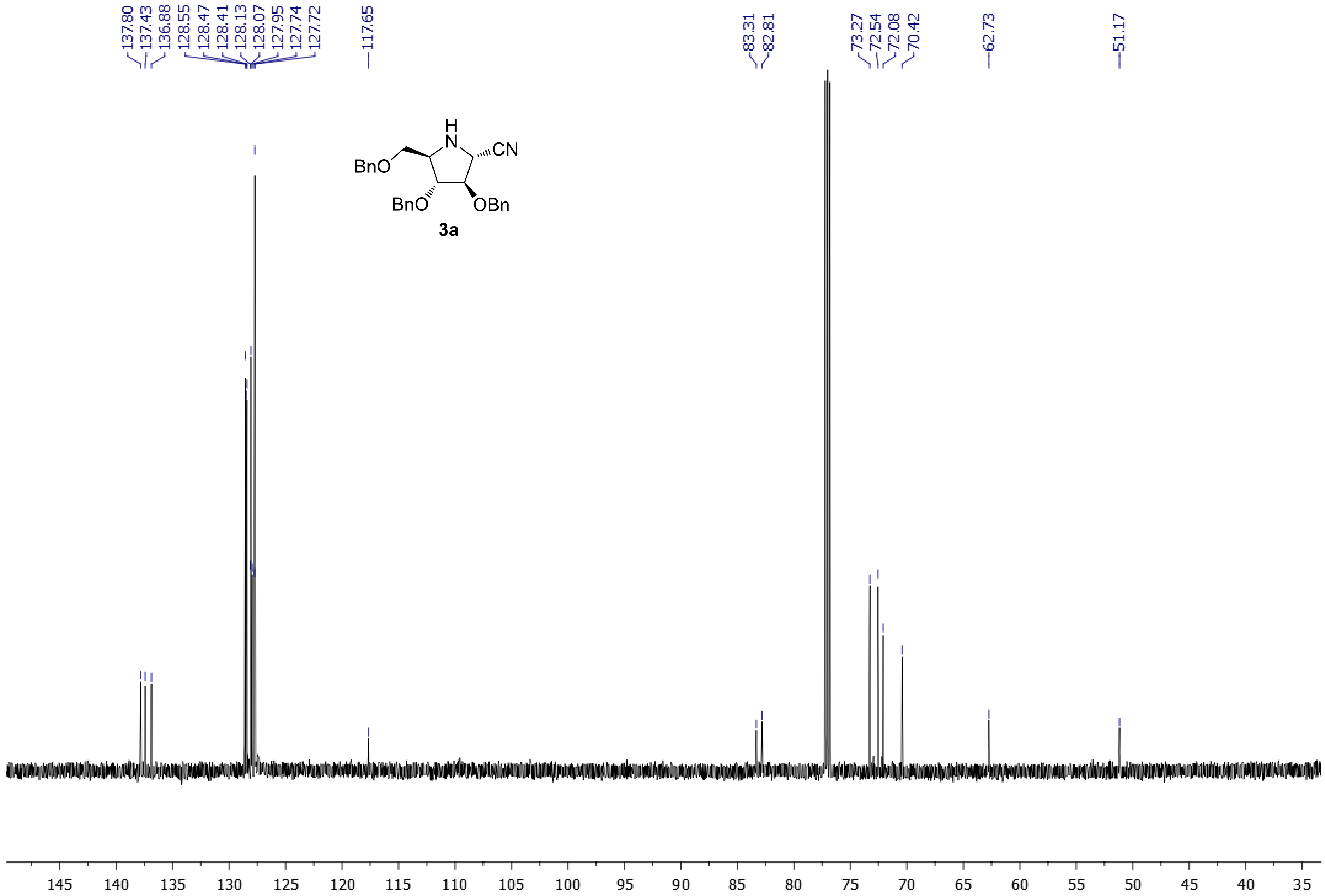
137.80
137.43
136.88
128.55
128.47
128.41
128.13
128.07
127.95
127.74
127.72
117.65
83.31
82.81
73.27
72.54
72.08
70.42
62.73
51.17
H
N
CN
BnO
BnO
OBn
3a
145 140 135 130 125 120 115 110 105 100 95 90 85 80 75 70 65 60 55 50 45 40 35

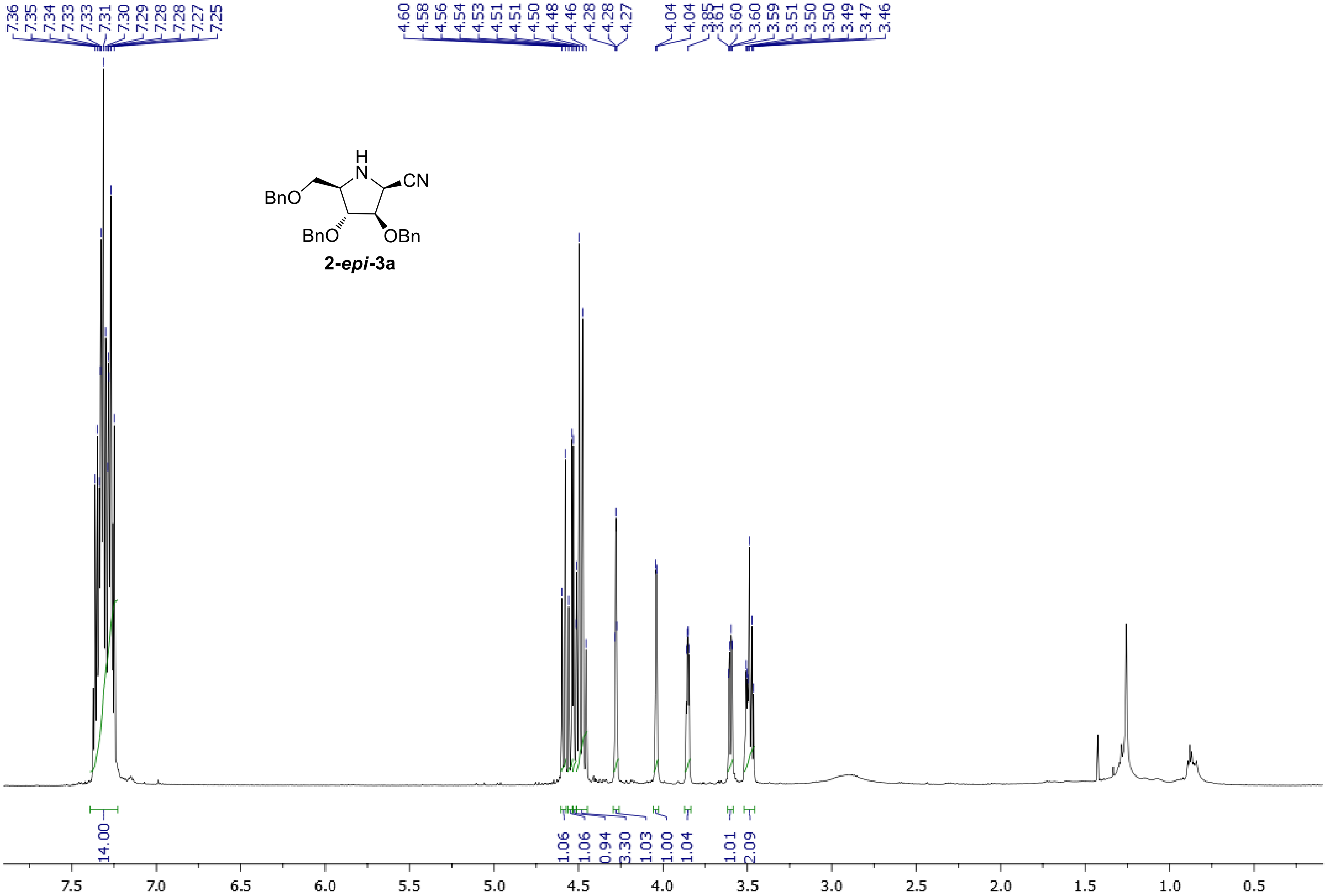

7.36 7.35 7.34 7.33 7.33 7.31 7.30 7.29 7.28 7.28 7.27 7.25
4.60 4.58 4.56 4.54 4.53 4.51 4.51 4.50 4.48 4.46 4.28 4.28 4.27
4.04 4.04 3.85 3.61 3.60 3.60 3.59 3.51 3.50 3.50 3.49 3.47 3.46
H
N
CN
BnO
BnO
OBn
2-epi-3a
14.00
1.06 1.06 0.94 3.30 1.03 1.00 1.04 1.01 2.09
7.5 7.0 6.5 6.0 5.5 5.0 4.5 4.0 3.5 3.0 2.5 2.0 1.5 1.0 0.5

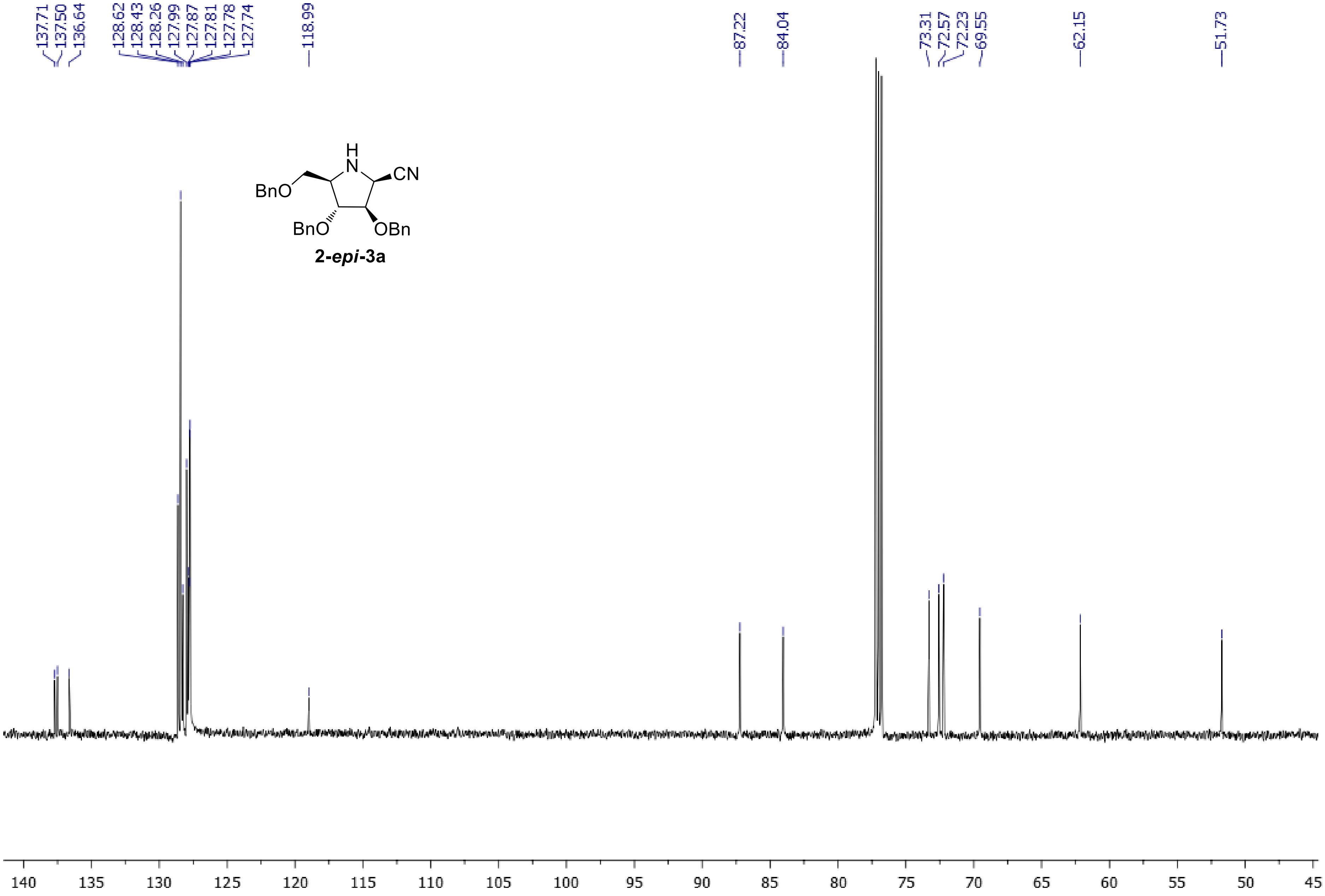
137.71
137.50
136.64
128.62
128.43
128.26
127.99
127.87
127.81
127.78
127.74
118.99
87.22
84.04
73.31
72.57
72.23
69.55
62.15
51.73
H
N
CN
BnO
BnO
OBn
2-*epi*-3a
140
135
130
125
120
115
110
105
100
95
90
85
80
75
70
65
60
55
50
45

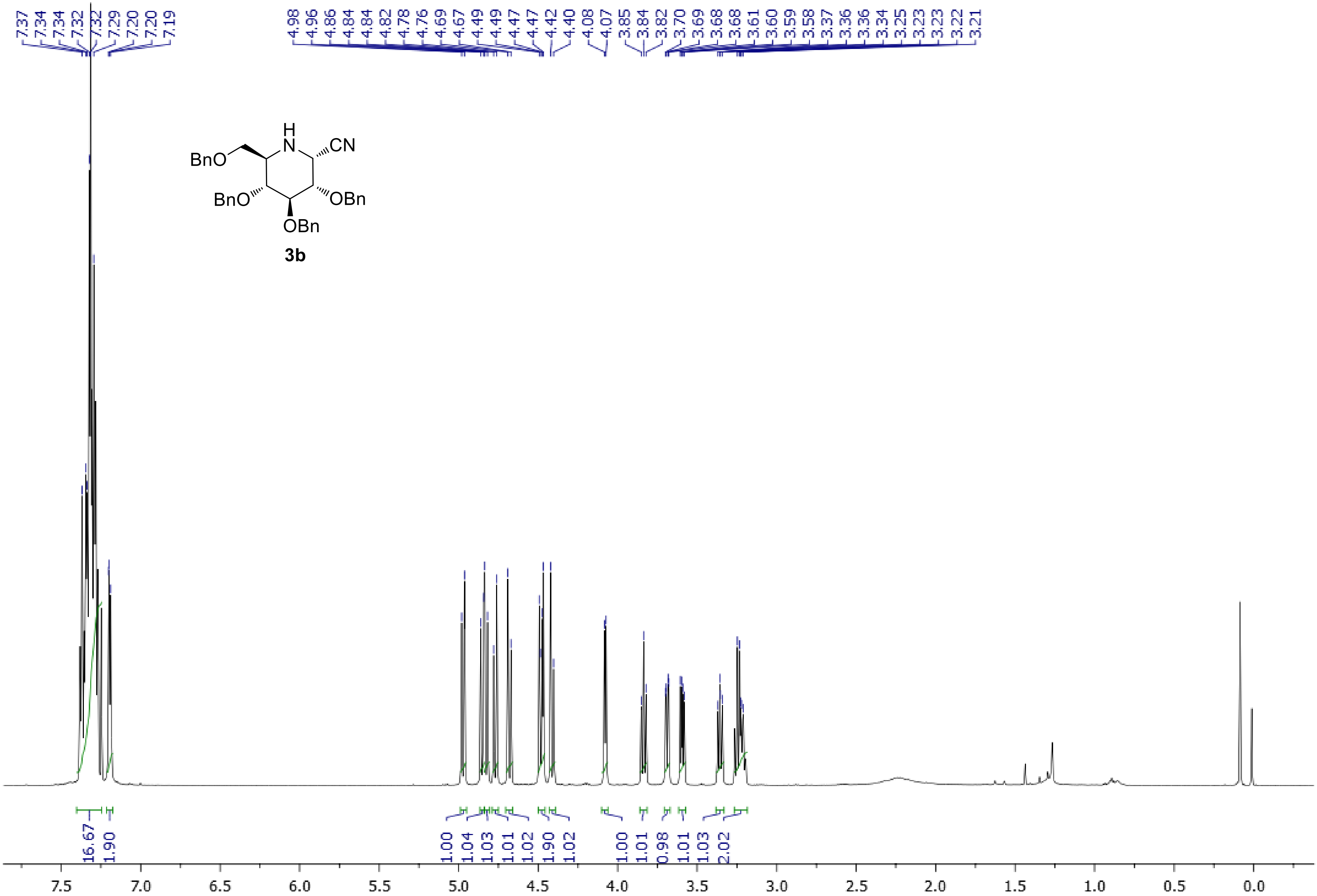
7.37 7.34 7.34 7.32 7.32 7.32 7.29 7.20 7.20 7.19
4.98 4.96 4.86 4.84 4.84 4.82 4.78 4.76 4.69 4.67 4.49 4.49 4.47 4.47 4.42 4.40 4.08 4.07 3.85 3.84 3.82 3.70 3.69 3.68 3.68 3.61 3.60 3.59 3.58 3.37 3.36 3.36 3.34 3.25 3.23 3.23 3.22 3.21
H
N
CN
BnO
BnO
OBn
OBn
3b
16.67 1.90
1.00 1.04 1.03 1.01 1.02 1.90 1.02
1.00 1.01 0.98 1.01 1.03 2.02
7.5 7.0 6.5 6.0 5.5 5.0 4.5 4.0 3.5 3.0 2.5 2.0 1.5 1.0 0.5 0.0

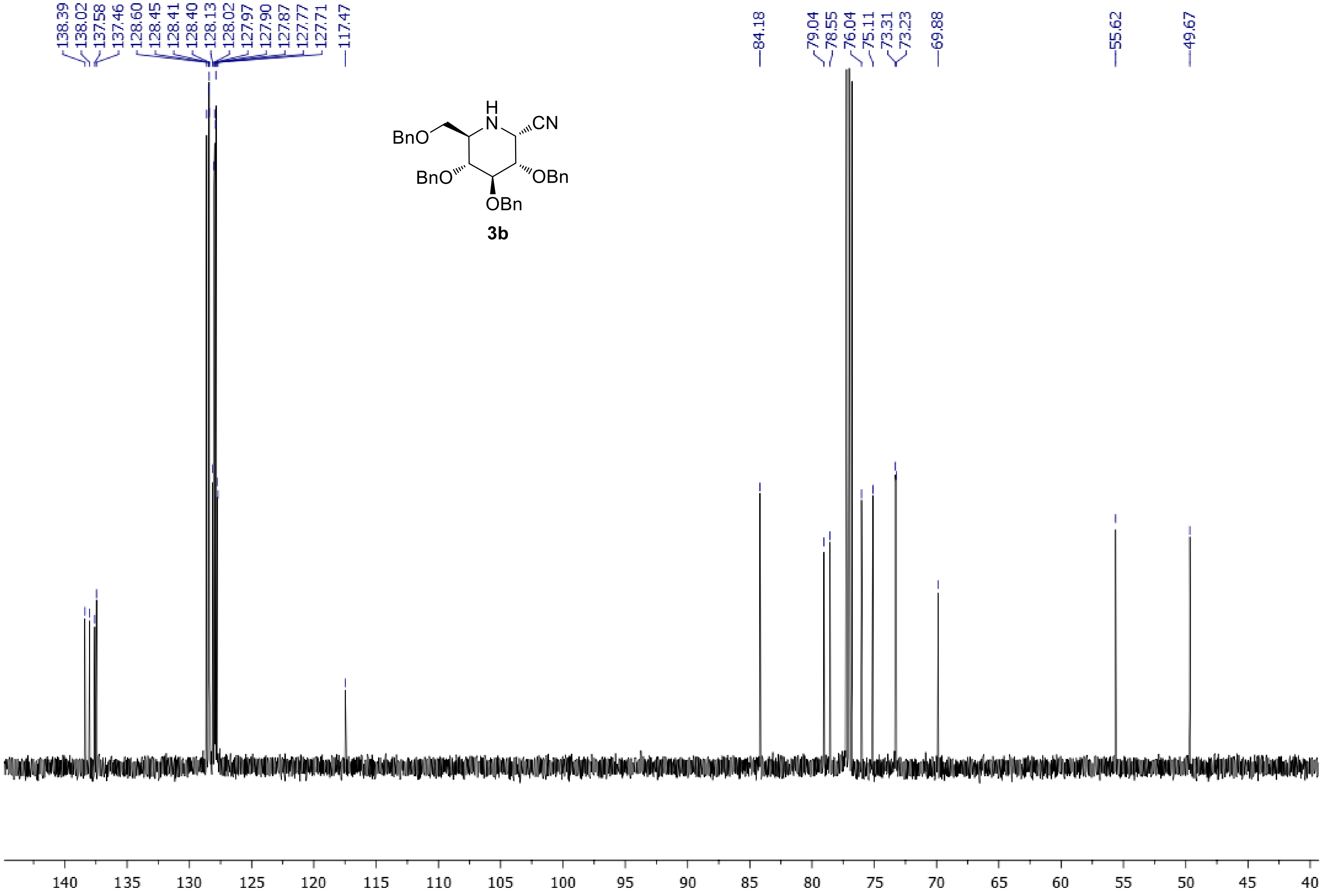
138.39
138.02
137.58
137.46
128.60
128.45
128.41
128.40
128.13
128.02
127.97
127.90
127.87
127.77
127.71
117.47
84.18
79.04
78.55
76.04
75.11
73.31
73.23
69.88
55.62
49.67
H
N
CN
BnO
BnO
OBn
OBn
3b
140 135 130 125 120 115 110 105 100 95 90 85 80 75 70 65 60 55 50 45 40

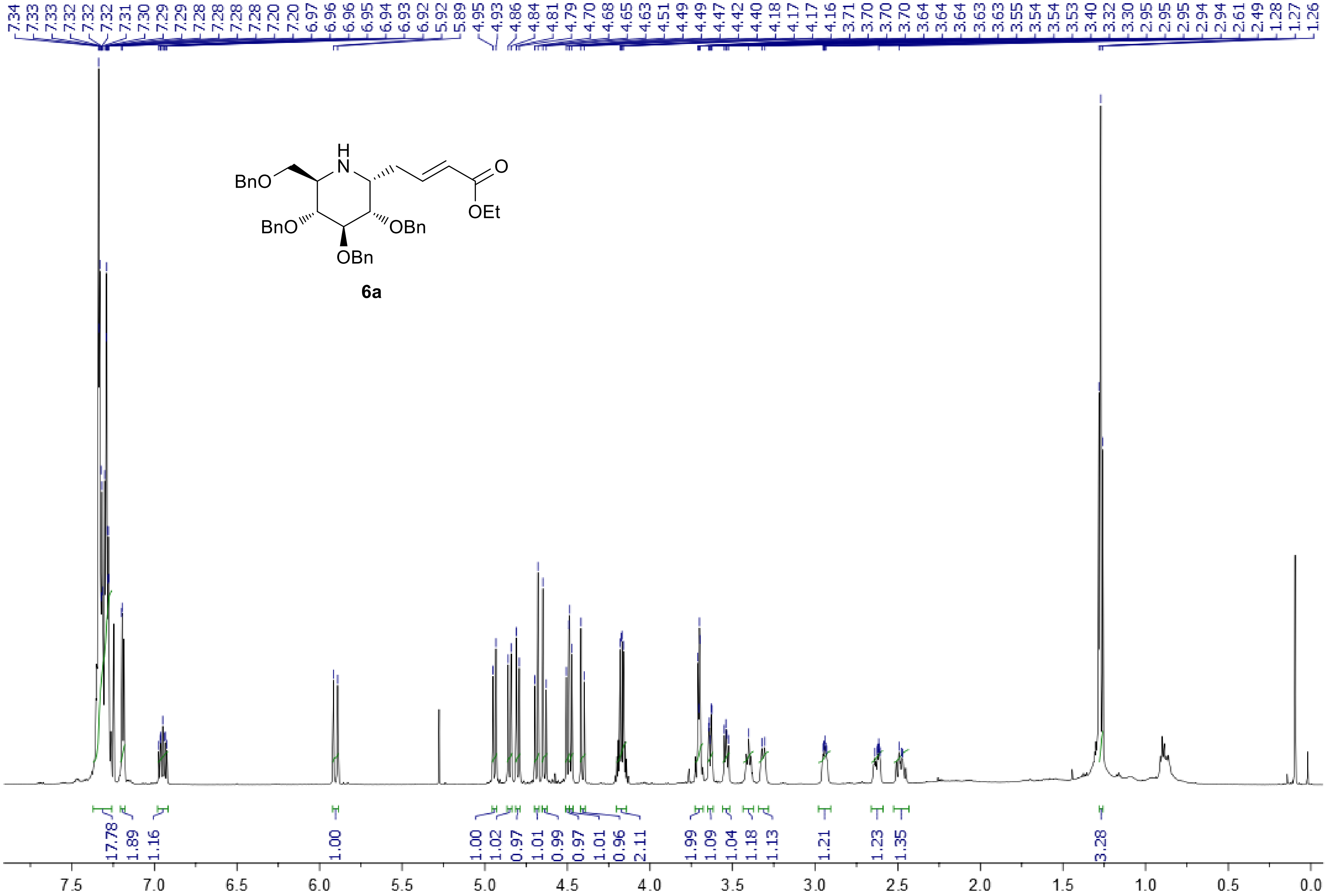

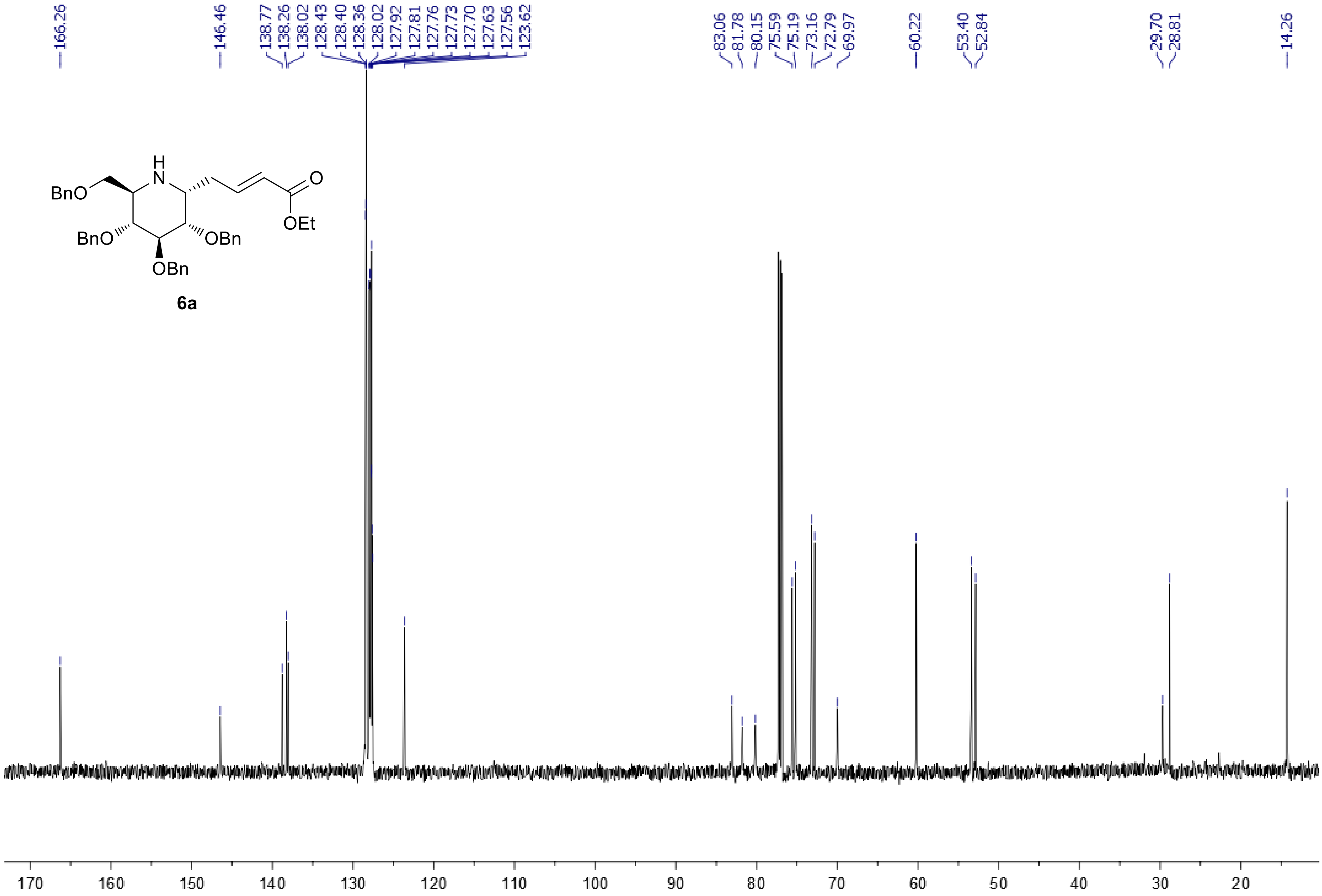
166.26
146.46
138.77
138.26
138.02
128.43
128.40
128.36
128.02
127.92
127.81
127.76
127.73
127.70
127.63
127.56
123.62
83.06
81.78
80.15
75.59
75.19
73.16
72.79
69.97
60.22
53.40
52.84
29.70
28.81
14.26
BnO
H
N
O
OEt
BnO
OBn
OBn
6a
170
160
150
140
130
120
110
100
90
80
70
60
50
40
30
20

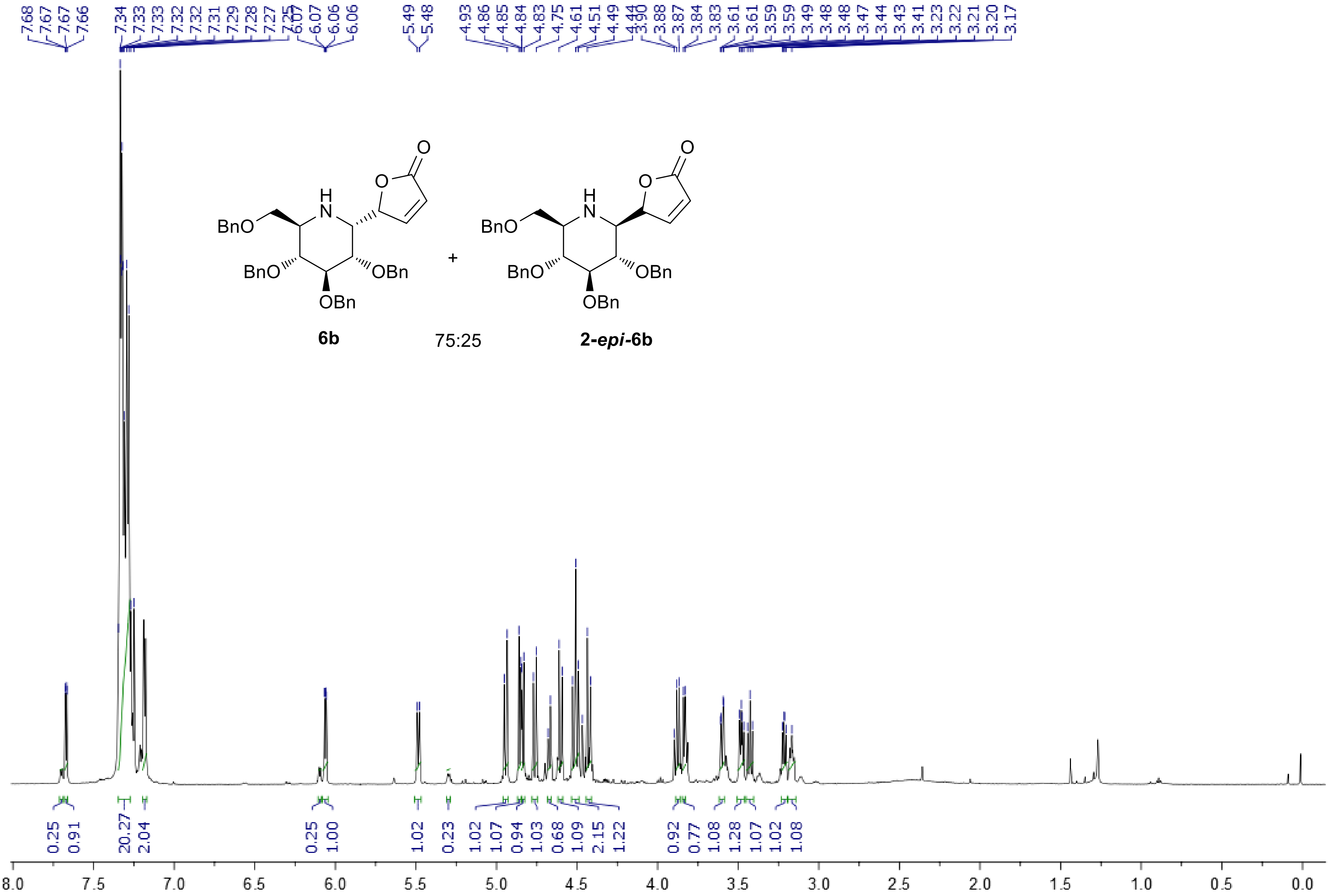

6b
75:25
2-*epi*-6b
BnO
OBn

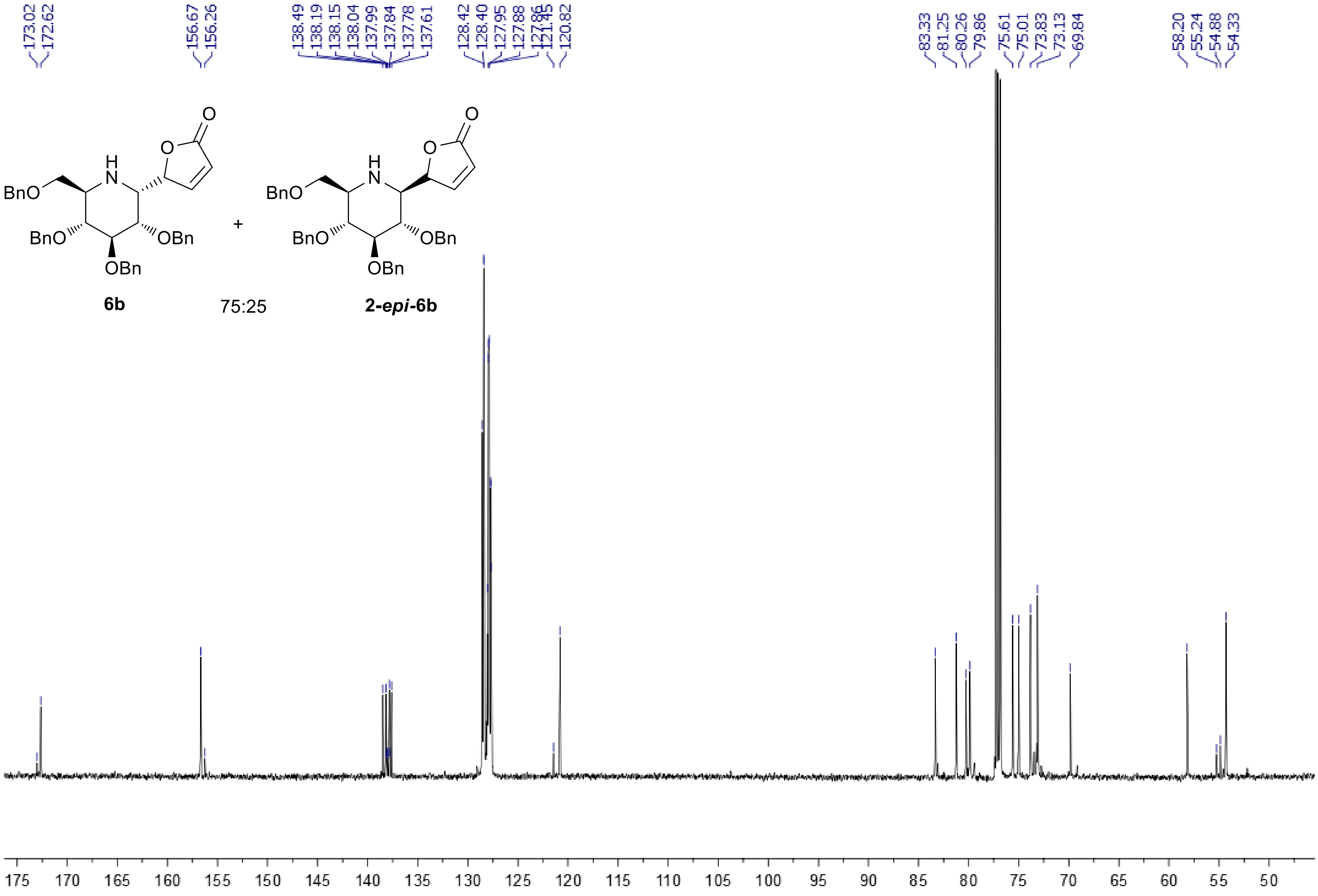
173.02
172.62
156.67
156.26
138.49
138.19
138.15
138.04
137.99
137.84
137.78
137.61
128.42
128.40
127.95
127.88
127.86
121.45
120.82
83.33
81.25
80.26
79.86
75.61
75.01
73.83
73.13
69.84
58.20
55.24
54.88
54.33
6b
75:25
2-epi-6b
175 170 165 160 155 150 145 140 135 130 125 120 115 110 105 100 95 90 85 80 75 70 65 60 55 50

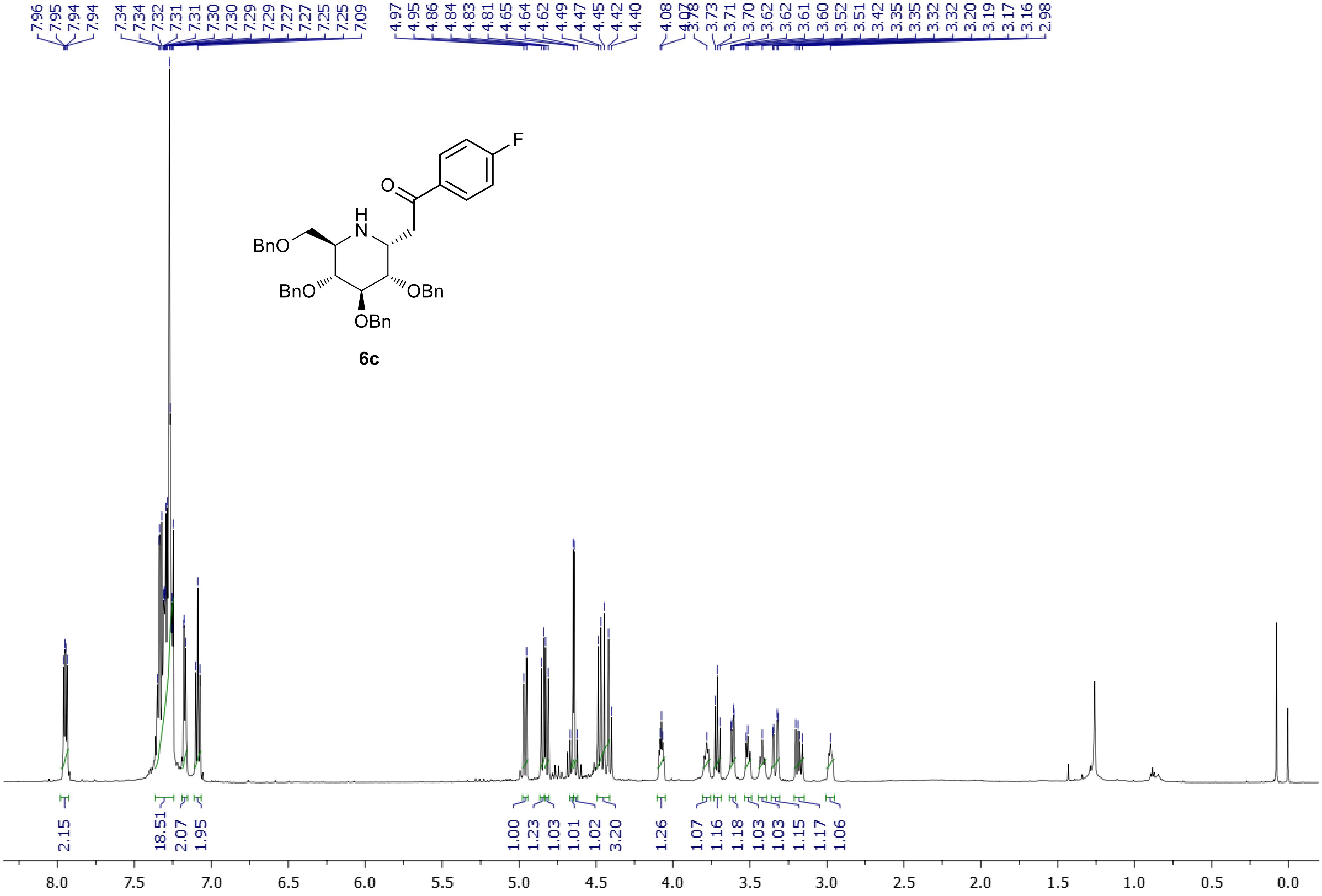
7.96 7.95 7.94 7.94 7.34 7.34 7.32 7.31 7.31 7.30 7.30 7.29 7.29 7.27 7.27 7.25 7.25 7.09 4.97 4.95 4.86 4.84 4.83 4.81 4.65 4.64 4.62 4.49 4.47 4.45 4.42 4.40 4.08 4.07 3.78 3.73 3.71 3.70 3.62 3.62 3.61 3.60 3.52 3.51 3.42 3.35 3.35 3.32 3.32 3.20 3.19 3.17 3.16 2.98
F
O
H
N
BnO
BnO
OBn
OBn
2.15 18.51 2.07 1.95 1.00 1.23 1.03 1.01 1.02 3.20 1.26 1.07 1.16 1.18 1.03 1.03 1.15 1.17 1.06
8.0 7.5 7.0 6.5 6.0 5.5 5.0 4.5 4.0 3.5 3.0 2.5 2.0 1.5 1.0 0.5 0.0

6c

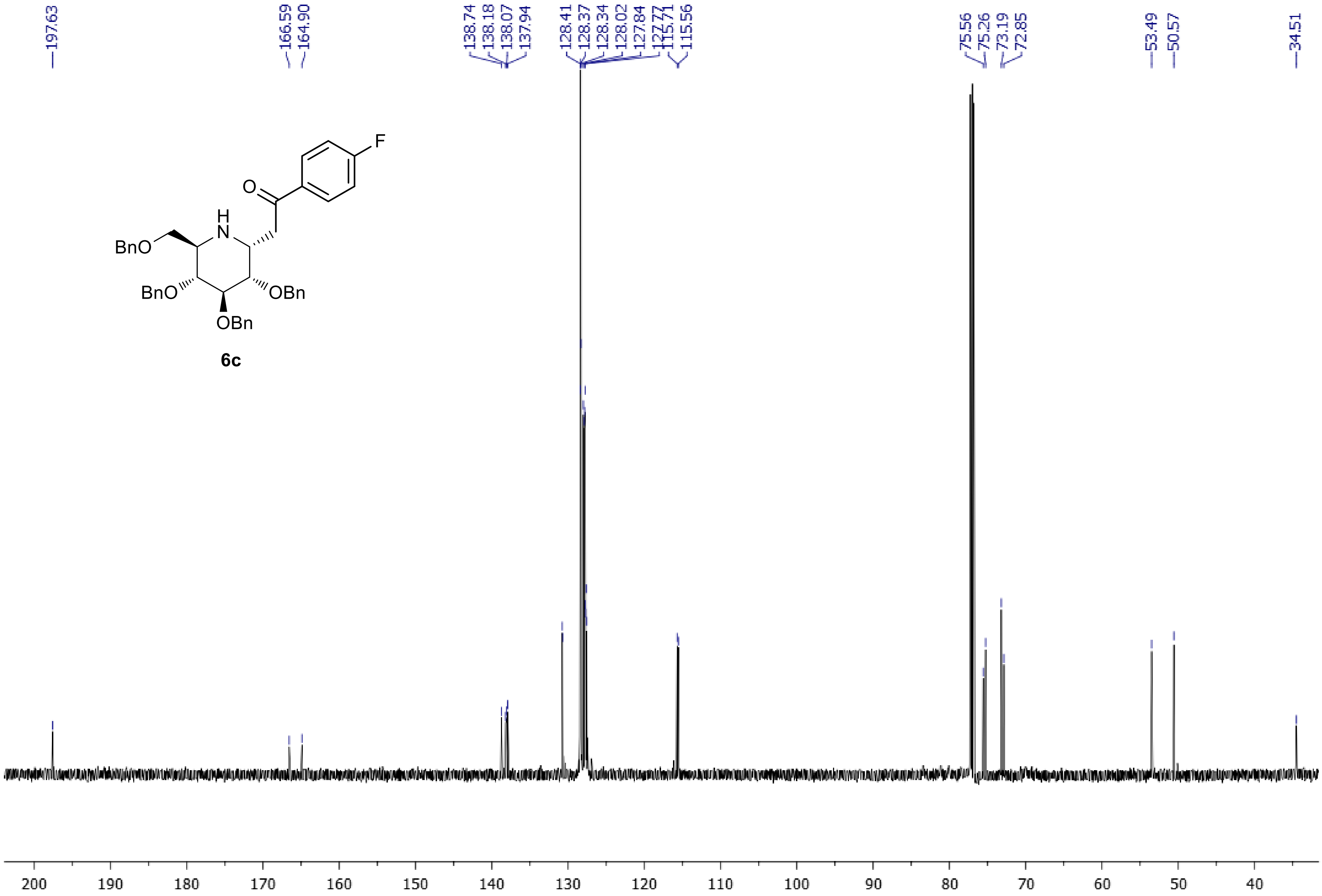

197.63
166.59
164.90
138.74
138.18
138.07
137.94
128.41
128.37
128.34
128.02
127.84
127.77
115.71
115.56
75.56
75.26
73.19
72.85
53.49
50.57
34.51
F
O
H
N
BnO
BnO
OBn
OBn
6c
200
190
180
170
160
150
140
130
120
110
100
90
80
70
60
50
40
